# Kidney transplantation from a living donor with renal artery fibromuscular dysplasia: A case report on arterial grafting of the donor renal artery

**DOI:** 10.1002/iju5.12188

**Published:** 2020-07-04

**Authors:** Yuto Matsushita, Daisuke Motoyama, Toshiki Ito, Takayuki Sugiyama, Atsushi Otsuka, Masaki Sano, Kazunori Inuzuka, Hideaki Miyake

**Affiliations:** ^1^ Department of Urology Hamamatsu University School of Medicine Hamamatsu Shizuoka Japan; ^2^ Department of Vascular Surgery Hamamatsu University School of Medicine Hamamatsu Shizuoka Japan

**Keywords:** arterial graft, fibromuscular dysplasia, living donor kidney transplantation

## Abstract

**Introduction:**

Renal artery fibromuscular dysplasia is generally considered a contraindication of kidney transplantation, since fibromuscular dysplasia occasionally induces hypertension or renal insufficiency in the recipient and/or donor. To date, limited information remains available with respect to whether kidneys with renal arterial fibromuscular dysplasia can be successfully transplanted.

**Case presentation:**

A 53‐year‐old potential donor was diagnosed with fibromuscular dysplasia of the right renal artery. Laparoscopic right nephrectomy was performed, and the affected portion was resected and reconstructed using the harvested internal iliac artery. Transplantation was successful and the serum creatinine level was <2 mg/dL for 3 years after surgery.

**Conclusion:**

If reconstruction of the diseased artery could be safely performed, transplantation using a kidney from a donor with renal artery fibromuscular dysplasia may be considered.

**Abbreviations & Acronyms**
CT: computed tomography
ERPF: effective renal plasma flow
FMD: fibromuscular dysplasia

**Keynote message**

Even if a kidney is received from a donor with FMD, kidney transplantation incorporating reconstruction of the affected renal artery could be successfully performed. However, careful long‐term follow‐up for both donors and recipients is necessary to ensure favorable renal function.

## Introduction

Renal artery FMD is an idiopathic disease accompanied by distorted architecture of the renal arterial wall.^1^ In potential kidney transplant donors, the incidence of renal artery FMD has been reported to be 2.0–6.6%.^2^, ^3^, ^4^, ^5^ Kidney transplantation from donors with FMD is generally regarded as a contraindication, because it may cause hypertension and renal insufficiency not only in the recipient but also in the donor. However, considering the chronic shortage of organ donations for renal transplantation, it is necessary to develop a safe strategy for the use of kidneys with renal artery FMD. Here, we describe a successful living donor renal transplantation using a kidney with renal artery FMD by incorporating the reconstruction of the artery.

## Case presentation

**Fig. 1:**
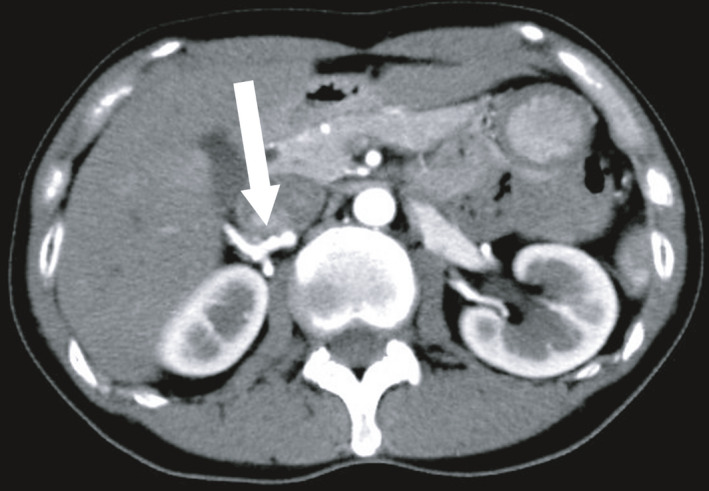
Contrast‐enhanced CT showing irregular lumen of the right renal artery (arrow).

**Fig. 2:**
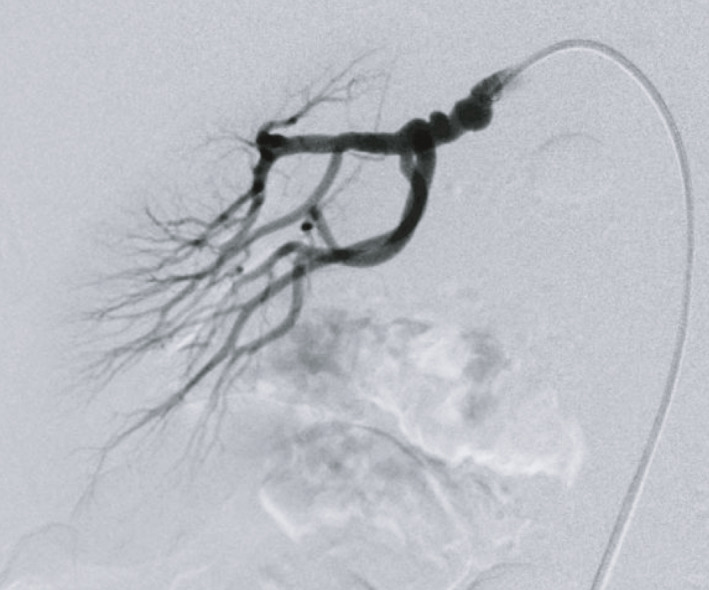
Renal arteriography revealing 15‐mm long string‐of‐beads pattern at the main right renal artery.

A 53‐year‐old woman was admitted to donate one of her kidneys to her husband. Contrast‐enhanced CT revealed irregular lumen in the right renal artery (Fig. 1); however, she had normal blood pressure and a blood test indicated normal renin activity and aldosterone level. Renal arteriography visualized a 15‐mm long string‐of‐beads pattern at the proximal right renal artery (Fig. 2), while the rest of the distal right renal artery and the whole left renal artery appeared intact. Based on these findings, she was diagnosed with moderate renal artery FMD. A renogram with technetium‐99m mercaptoacetyltriglycine revealed that the ERPFs of the right and left kidneys were 48.4 and 64.1 mL/min, respectively. Considering the laterality of FMD as well as the difference of ERPF, her right kidney was judged to be suitable as a graft kidney.

**Fig. 3:**
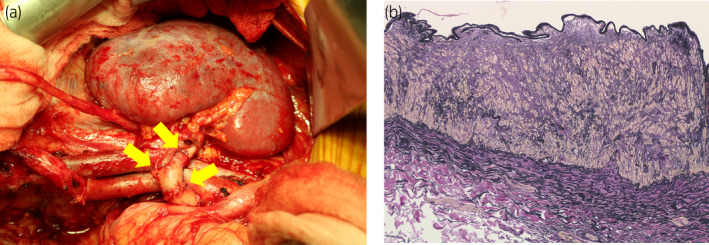
(a) The renal artery was reconstructed by a graft of the internal iliac artery and its brunch harvested from the recipient. End‐to‐end anastomosis was performed using a single‐stitch technique (arrows). (b) Elastica van Gieson staining of the right renal artery indicated fibroplasia in the arterial medium and proliferation of smooth muscle tissue cells.

Laparoscopic right donor nephrectomy by the retroperitoneal approach and kidney transplantation to the recipient were performed. Following the removal of the donor kidney, we resected 20 mm of the right renal artery, including 15 mm of the diseased portion documented by preoperative imaging examinations, considering the finding of intraoperative palpation. The renal artery was then reconstructed using a graft of the internal iliac artery and its brunch harvested from the recipient on extracorporeal surgery. As shown in Figure 3a, end‐to‐end anastomosis between the donor renal artery and arterial graft was performed at two proximal sites using the interrupted suture. Then, the graft kidney was transplanted in the right iliac fossa by vascular anastomoses with arterial graft to the internal iliac artery in an end‐to‐end fashion and the renal vein to the external iliac vein in an end‐to‐side fashion. In this case, the warm ischemia time and cold ischemia time were 3 and 147 min, respectively. Affected artery was pathologically diagnosed with medial‐type FMD, characterized by fibroplasia in the arterial medium and proliferation of smooth muscle tissue cells (Fig. 3b).

After transplantation, the subsequent courses in both the donor and recipient were surgically unremarkable, and the renal graft functioned favorably with a serum creatinine level of 1.6 mg/dL, resulting in the withdrawal of hemodialysis. The renal graft function was stable with a serum creatinine level of <2 mg/dL for 3 years after transplantation in the recipient, and blood pressure in both the donor and recipient was normal without medication.

## Discussion

FMD is a non‐atherosclerotic disease of the musculature of arteries accompanied by abnormal cellular proliferation. To diagnose FMD, computed tomographic angiography is initially performed; however, it is necessary to conduct catheter‐based angiography to obtain precise findings on location and morphological features. Despite being frequently asymptomatic, FMD can cause renovascular hypertension and is managed by medication against hypertension, angioplasty by catheter, and/or surgery.^1^

The prevalence of renal artery FMD is 2.0–6.6% of the potential donor population.^2^, ^3^, ^4^, ^5^ However, the guidelines for living kidney donors developed in the Amsterdam Forum did not mention the eligibility of donor candidates with FMD.^6^ Although renal artery FMD is usually regarded as a contraindication of kidney transplantation considering the potential risks of postoperative hypertension and renal insufficiency in both the donor and recipient, there have been reports of successful cases. Kolettis *et al*. conducted a retrospective review of a departmental database of renal transplantations, and identified 36 donors with FMD. After transplantation, recipients from these donors achieved functional outcomes similar to those from non‐FMD donors under adequate preoperative assessment including arteriography. Based on these findings, they concluded that selected patients with renal artery FMD who showed normal blood pressure and medial‐type FMD could be donors for renal transplantation.^7^ Pfeiffer *et al*. also found that even kidneys from donors with severe renal artery FMD could be successfully transplanted, if the affected renal artery segments could be precisely reconstructed.^8^

In our case, although moderate FMD was present at the proximal right renal artery, it was assumed that the affected segment could be reconstructed by arterial grafting according to the assessments of preoperative examinations. FMD was only detected unilaterally, and the contralateral renal artery lacked signs of FMD; therefore, the removal of the affected kidney by FMD could result in a reduced risk of future development of hypertension in the donor. Considering these findings, we decided to transplant the kidney. Although Pfeiffer *et al*. recommended vein graft replacement as the most suitable method,^8^ considering the comparatively high perioperative risk of bleeding complications after reconstruction of the renal artery with a harvested vein, arterial replacement could be a safe alternative in such cases. Indeed, despite being an initial case using the internal iliac artery as a graft vessel, this case exhibited a favorable perioperative course without complications.

Although there were some studies showing the contralateral kidney remained free from FMD in patients donated the affected kidneys,^9^, ^10^ we would like to emphasize the importance of a long‐term careful follow‐up for both the donor and recipient after renal transplantation, considering the possibility of developing postoperative FMD. Parasuraman *et al*. reported a living kidney donor affected by renovascular hypertension 1 year after donation. She was not diagnosed with FMD before donation, but retrospective careful review of preoperative CT indicated very mild arterial stenosis.^11^ Bonatti *et al*. also described a donor affected with hypertension and renal dysfunction by the FMD 8 years after donation.^12^ In addition to living donor transplantation, several cases after deceased donor transplantation were treated with percutaneous transluminal angioplasty.^13^, ^14^, ^15^, ^16^ Due to the difficulty of preoperative precise assessment, intensive observation is important particularly after renal transplantation from deceased donor.

In this report, we described a successfully performed living donor renal transplantation from a donor with moderate FMD of the renal artery by reconstructing the affected artery using the internal iliac artery harvested from the recipient. Based on this case, the use of kidneys from donors with FMD should be considered, resulting in a contribution to increase the donor pool and to decrease waiting time for renal transplantation. However, it should be recognized that the transplantation of a kidney from a donor with FMD is a technically challenging operation requiring vascular surgical expertise; thus, collaboration with a vascular surgeon may be necessary in some institutions.

## Conflict of interest

The authors declare no conflict of interest.
